# Editorial: Recent advances, new perspectives and applications of conceptual density functional theory

**DOI:** 10.3389/fchem.2022.1003106

**Published:** 2022-09-09

**Authors:** Norma Flores-Holguín, Juan Frau, Daniel Glossman-Mitnik

**Affiliations:** ^1^ Laboratorio Virtual NANOCOSMOS, Departamento de Medio Ambiente y Energía, Centro de Investigación en Materiales Avanzados, Chihuahua, Chih, Mexico; ^2^ Departament de Química, Universitat de Les Illes Balears, Palma de Mallorca, Spain

**Keywords:** density functional theory (DFT), conceptual DFT (CDFT), chemical reactivity, molecular descriptors, drug design and discovery, catalysis, alternative energy applications

Density Functional Theory (or DFT, for short) is a potent methodology useful for the calculation of the molecular and electronic structure of atoms, molecules, clusters, and solids, both in gas phase or in solution (Parr and Yang, 1989). The research in this area goes from the discovery of new computational methods based on the design of accurate density functionals to the application of this methodology for the prediction of the chemical and physical properties of the studied systems. Its use relies not only in the ability to calculate the molecular properties of the species of interest, but also in providing interesting concepts that allow a better comprehension of the chemical reactivity of the systems under consideration (Glossman-Mitnik, 2019; Ramasami, 2019; De Lazaro et al., 2021).

Conceptual Density Functional Theory (or CDFT, for short) is a branch of DFT based on the goal of developing a chemical reactivity theory founded on DFT-based concepts with the electron density being the starting point for the design of several response functions, or descriptors, that could be of help not only in the interpretation of the chemical reactivity but also in the predictions of properties and phenomena not having accessed experimentally (Chermette, 1999; Geerlings et al., 2003; Gázquez, 2009; Domingo et al., 2016). CDFT entwines mathematical and physical rigor with qualitative chemical intuition and its scope focuses on many broad applications across all branches of chemistry, from inorganic and materials chemistry, to organic chemistry, polymer chemistry, biochemistry, catalysis and nanochemistry (Chattaraj, 2009; Poater et al., 2010; Domingo et al., 2016; Geerlings et al., 2020; Chattaraj and Chakraborty, 2021; Glossman-Mitnik, 2022; Liu, 2022). Of paramount importance for the field of Conceptual DFT is the research work developed by Geerlings et al. (2020), which are contributing with two manuscripts in this Frontiers Research Topic, and which have presented in the past, among a vast number of publications, some very interesting research related with variational principles for describing chemical reactions through condensed reactivity indices (Ayers et al., 2002), the understanding of the Woodward–Hoffmann rules by using changes in the electron density (Ayers et al., 2007) and the quantitative correlation of several theoretical electrophilicity measures over different families of organic compounds (González-Martínez et al., 2010).

This Research Topic aims to include examples on some recent advances, new perspectives, and current and potential applications of CDFT for the understanding of the chemical reactivity of useful systems, as well as comprehensive overviews of the current scientific developments in this important field, by considering the fact that a chemical reaction will only occur when the reagent encounters a suitable reaction partner. Miranda-Quintana et al. developed reactivity rules that depend on both reagents to determine whether a pair of reagents is well-matched, resulting in a nice contribution to the fundamentals of Conceptual DFT. A continuation of this research was endowed by Miranda-Quintana et al. who studied the molecular interactions from the Density Functional Theory for chemical reactivity and derived an expression for the interaction energy between two reagents in terms of the chemical reactivity indicators that can be derived from density functional perturbation theory. Their work gives new support for the explanation of reactivity in terms if the “‖dμ‖ big is good” rule and the maximum hardness principle.

As activated Cdc42-associated kinase 1 (ACK1/TNK2) has a significant role in cell endocytosis, survival, proliferation, and migration, Srivastava employed Conceptual DFT and ADMET predictions for the study of the chemical reactivity and optical and pharmacokinetics properties of 14 multikinase inhibitors as well as the determination of their docking interactions towards ACK1 for precision oncology. By exploiting the capacity of the Dual Descriptor DD to reveal sites susceptible to undergo attacks simultaneously of nucleophilic and electrophilic types, and considering the advantage of DD of being an orbital-free descriptor, Martínez-Araya studied the Janus-faced ligand behavior of diiodine (I_2_). This work represented an application of the generalized operational formula of the dual descriptor that takes into account any possible degeneracy in Frontier molecular orbitals.

Rangel-Galván et al. considered Conceptual DFT and QTAIM analyses were performed to theoretically characterize the chemical reactivity properties and the structural stability of conformations of anandamide, which is a relevant biological ligand due to its capacity of interacting with several proteins, including the T-type calcium channels, which play an important role in neuropathic pain and depression disorders. A detailed characterization of the chemical properties and conformational stability of anandamide was performed to provide valuable information to understand its behavior in a biological context. Quezada-Borja et al. worked on the design of new hole transport materials based on triphenylamine derivatives using different μ-linkers for the application in perovskite solar cells. The materials were studied using DFT and Tight-Binding DFT and the results show they could be considered as new hole transport materials (HTMs) for their use in perovskite solar cells.

The objective of the collaboration between colleagues, which is one of the purposes of the Frontiers Research Topics, has been achieved within this Research Topic by showing the importance of the results obtained by means of DFT and Conceptual DFT, both from a theoretical point of view as well as from different applications.
